# Overview of Synthetic Cannabinoids ADB-FUBINACA and AMB-FUBINACA: Clinical, Analytical, and Forensic Implications

**DOI:** 10.3390/ph14030186

**Published:** 2021-02-25

**Authors:** Carolina Lobato-Freitas, Andreia Machado Brito-da-Costa, Ricardo Jorge Dinis-Oliveira, Helena Carmo, Félix Carvalho, João Pedro Silva, Diana Dias-da-Silva

**Affiliations:** 1 UCIBIO-REQUIMTE, Laboratory of Toxicology, Department of Biological Sciences, Faculty of Pharmacy, University of Porto, 4050-313 Porto, Portugal; carolinafreitas97@hotmail.com (C.L.-F.); ricardo.dinis@iucs.cespu.pt (R.J.D.-O.); helenacarmo@ff.up.pt (H.C.); felixdc@ff.up.pt (F.C.); 2 IINFACTS-Institute of Research and Advanced Training in Health Sciences and Technologies, Department of Sciences, University Institute of Health Sciences (IUCS), CESPU, CRL, 4585-116 Gandra, Portugal; andreia.machado2403@gmail.com; 3 Department of Public Health and Forensic Sciences, and Medical Education, Faculty of Medicine, University of Porto, 4200-319 Porto, Portugal

**Keywords:** synthetic cannabinoids (SCs), psychoactive substances, metabolism, toxicity, intoxication

## Abstract

ADB-FUBINACA and AMB-FUBINACA are two synthetic indazole-derived cannabinoid receptor agonists, up to 140- and 85-fold more potent, respectively, than *trans*-∆^9^-tetrahydrocannabinol (∆^9^-THC), the main psychoactive compound of cannabis. Synthesised in 2009 as a pharmaceutical drug candidate, the recreational use of ADB-FUBINACA was first reported in 2013 in Japan, with fatal cases being described in 2015. ADB-FUBINACA is one of the most apprehended and consumed synthetic cannabinoid (SC), following AMB-FUBINACA, which emerged in 2014 as a drug of abuse and has since been responsible for several intoxication and death outbreaks. Here, we critically review the physicochemical properties, detection methods, prevalence, biological effects, pharmacodynamics and pharmacokinetics of both drugs. When smoked, these SCs produce almost immediate effects (about 10 to 15 s after use) that last up to 60 min. They are rapidly and extensively metabolised, being the *O*-demethylated metabolite of AMB-FUBINACA, 2-(1-(4-fluorobenzyl)-1*H*-indazole-3-carboxamide)-3-methylbutanoic acid, the main excreted in urine, while for ADB-FUBINACA the main biomarkers are the hydroxdimethylpropyl ADB-FUBINACA, hydroxydehydrodimethylpropyl ADB-FUBINACA and hydroxylindazole ADB-FUBINACA. ADB-FUBINACA and AMB-FUBINACA display full agonism of the CB1 receptor, this being responsible for their cardiovascular and neurological effects (e.g., altered perception, agitation, anxiety, paranoia, hallucinations, loss of consciousness and memory, chest pain, hypertension, tachycardia, seizures). This review highlights the urgent requirement for additional studies on the toxicokinetic properties of AMB-FUBINACA and ADB-FUBINACA, as this is imperative to improve the methods for detecting and quantifying these drugs and to determine the best exposure markers in the various biological matrices. Furthermore, it stresses the need for clinicians and pathologists involved in the management of these intoxications to describe their findings in the scientific literature, thus assisting in the risk assessment and treatment of the harmful effects of these drugs in future medical and forensic investigations.

## 1. Introduction

Synthetic cannabinoids (SCs), also known as synthetic cannabinoid receptor agonists (SCRAs), represent the largest group of new psychoactive substances (NPS) currently monitored by the European Monitoring Centre for Drugs and Drug Addiction (EMCDDA) through the EU Early Warning System [1]. Many of the already identified SCs have been involved in numerous cases of poisonings and deaths [2,3,4,5]. Most of these recreational substances were originally synthesised for biomedical and therapeutic research, but currently there are several laboratories, mainly in China, that produce and export them in bulk powder to Europe [6,7]. These products are locally dissolved in organic solvents and subsequently sprayed over dry plant matter to cause the misleading impression of being as natural as cannabis; or encapsulated for oral consumption [6,7].

In 2009, the pharmaceutical company Pfizer Inc. patented the compound (*S*)-*N*-(1-amino-3,3-dimethyl-1-oxobutan-2-yl)-1-(4-fluorobenzyl)-1*H*-indazole-3-carboxamide (ADB-FUBINACA), which was developed to act as a potential therapeutic agent for disorders mediated by the type-1 cannabinoid receptor (CB1R) [8]. In 2013, ADB-FUBINACA was first identified in Japan in mixtures of herbs and other NPS (e.g., α-pyrrolidinopentiothiophenone and AH-7921) for recreational use [9]. That same year, this substance was first detected in Europe, specifically in Hungary, both in pills labelled with a Facebook logo, and in biological samples of consumers [10]. In 2014, the ADB-FUBINACA analogue methyl (*S*)-2-[1-(4-fluorobenzyl)-1*H*-indazole-3-carboxamido]-3-methylbutanoate (AMB-FUBINACA), also known as FUB-AMB and MMB-FUBINACA, was first detected in the state of Louisiana, USA, and in Sweden; emerging in the city of Auckland, New Zealand, in 2017, and having recently gained great notoriety in different parts of the world [1,2,11,12].

ADB-FUBINACA and AMB-FUBINACA are among the most widely abused and seized NPS [2,5,13] and similarly to other SCs, they are mainly marketed over the internet as more potent substitutes of cannabis [1,14]. Being synthetic agonists with a greater affinity, potency and efficacy for CB1R and type-2 cannabinoid receptor (CB2R) than *trans*-∆^9^-tetrahydrocannabinol (∆^9^-THC, the main psychoactive component of cannabis) [15,16,17], activation of these receptors by ADB-FUBINACA and/or AMB-FUBINACA produces more intense psychotropic effects and increased severity of the cardiovascular and neurological effects, compared to ∆^9^-THC, even when the drugs are consumed in smaller amounts [3,18,19,20,21]. Among the toxic effects elicited by ADB-FUBINACA and AMB-FUBINACA, it is worth mentioning the severe changes in mental status, the occurrence of seizures, fever, cardiotoxicity, rhabdomyolysis, kidney damage, and ultimately death [22,23,24,25]. However, knowledge of the pharmacological and toxicological mechanisms of ADB-FUBINACA and AMB-FUBINACA remains limited.

Since these SCs display a molecular structure different from that of ∆^9^-THC, their detection is often challenging, as they will not be spotted by the existing tests for screening of cannabis consumption. Moreover, as they are generally extensively metabolised, the concentration of parent compound detected in urine after consumption is usually very low or absent. For these reasons, it has been difficult to document ADB-FUBINACA and AMB-FUBINACA consumption in forensic and clinical cases, as well as to diagnose and treat intoxications, which is currently based on symptomatic improvement. In addition, as these substances are not normally consumed separately, but in combination with other drugs, the above-mentioned difficulties are further increased [2,10,26].

This review summarises the available information on ADB-FUBINACA and AMB-FUBINACA regarding their physicochemical properties and detection methods, abuse and prevalence patterns, legal status, biological and clinical effects, mechanisms of toxicity and treatment of intoxications, particularly focusing on their pharmacodynamics and pharmacokinetics. In this sense, this work intends to alert all readers, including clinicians and pathologists, and the regulatory authorities for the risks associated with the use of these substances. In addition, it intends to provide data that allows to (i) guide future experimental plans for toxicological and pharmacological research, (ii) detect at early stages and with greater rigor the involvement of such substances in severe and fatal intoxications, as well as in apprehended products, and (iii) select the best therapeutic strategies to be adopted in case of ADB-FUBINACA and/or AMB-FUBINACA-related intoxications.

## 2. Methodology

A bibliographic search was carried out using the PubMed (National Library of Medicine of the USA) database, considering papers published until December 2020. Only articles written in English were considered. Using the terms “AMB-FUBINACA”, “FUB-AMB” or “MMB-FUBINACA”, a total of 31 articles were found, while the term “ADB-FUBINACA” retrieved 27 articles. Additionally, documents from regulatory agencies such as the EMCDDA, World Health Organization (WHO) and Drug Enforcement Administration (DEA) were consulted to obtain additional information about these SCs. The entire bibliography of these documents and publications was rigorously explored to find additional publications relevant to this review. In total, 154 articles were analysed.

## 3. Chemistry and Chemical Analysis

**Figure 1:**
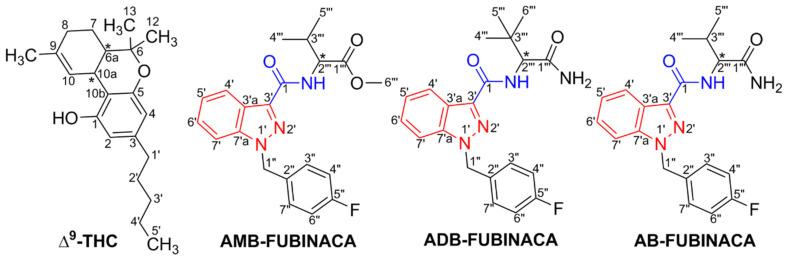
Comparison of the molecular structures of synthetic cannabinoid receptor agonists with that of *trans*-∆^9^-tetrahydrocannabinol (∆^9^-THC). The indazole core is represented in red and the carboxamide link in blue.

(*S*)-*N*-(1-Amino-3,3-dimethyl-1-oxobutane-2-yl)-1-(4-fluorobenzyl)-1*H*-indazole-3-carboxamide (ADB-FUBINACA) and methyl (*S*)-2-[1-(4-fluorobenzyl)-1*H*-indazole-3-carboxamido]-3-methylbutanoate (AMB-FUBINACA) comprise an indazole core structure (red) featuring an amide group (blue) at the 3-position (Figure 1), thus belonging to the group of indazole-carboxamide SCs [18,27]. The indazole skeleton is thought to help stabilise the binding of these SCs to the CB1R [28].

ADB-FUBINACA is the methylated analogue of the SC AB-FUBINACA (Figure 1), which was also developed and patented (patent reference: WO 2009/106980-A2) by Ingrid Buchler and her colleagues at Pfizer Inc. in 2009, having never been tested in humans [8,29]. From the data revealed in the ADB-FUBINACA patent, it is likely that the cannabimimetic activity of ADB-FUBINACA is produced by the (*S*)-enantiomer (chiral carbon in C-2). Although the stereochemistry of ADB-FUBINACA is not fully determined, the existence of an *(R*)-ADB-FUBINACA enantiomer is plausible [8,20]. AMB-FUBINACA has also the (*S*)- and (*R*)-configurations and the chiral centre at the C-2 carbon of the valinate side chain [29]. Chirality is common among SCs, implying different pharmacological and toxicological potencies among enantiomers [30,31,32,33,34]. As such, the assessment of the proportions of (*S*)- and (*R*)-enantiomers in the seized materials may allow for a better estimate of the risks users are exposed to. Antonides et al. [31] analysed sized herbal products, reporting a predominance of the (*S*)-enantiomer of AMB-FUBINACA in all samples (96.8 to 98.2%), which is in line with studies revealing the (*S*)-enantiomer of other SCs as the most prevalent or the only detected in illicit herbal products [30]]. Of note, the (*S*)-enantiomer is generally more potent than the (*R*)-enantiomer in both CB1R and CB2R [30,32,34], the potency varying amongst SCs [31,33]. In this line, (*S*)-AMB-FUBINACA has greater affinity for both CB1R (*S/R* ratio of 6.13) and CB2R (*S/R* ratio of 1.55), than (*R*)-AMB-FUBINACA [31]. For CB2R, the relative potency of *S/R* was shown to be impacted by structural characteristics, the difference being more prominent for compounds with an amine moiety than compounds with an ester moiety (such as AMB-FUBINACA, which explains the low CB2R *S/R* ratio) [31]. The potency in the (*R*)-enantiomer increases for SCs displaying the configuration of valinate methyl ester, at the detriment of valinamide, leucinamide or tert-leucine methyl ester. In addition, the efficacy of the (*S*)-AMB-FUBINACA was also found to be almost two times greater in CB1R, with a maximal response (E_max_) of 267% for the (*S*)-enantiomer and 154% for the (*R*)-enantiomer, as compared to the control (JWH-018); while the efficacy in CB2R was lower for the (*S*)-enantiomer (E_max_ of 161%), when compared to the (*R*)-enantiomer (E_max_ of 205%) [31,33,34].

AMB-FUBINACA has two absorbance peaks in the UV-Vis spectrum, at 208 nm and 299 nm [29], while the UV spectrum of ADB-FUBINACA shows a peak at 302 nm [9]. The main physicochemical properties of ADB-FUBINACA and AMB-FUBINACA are summarised in Table 1 [20,35,36].

The spectroscopic characteristics of ADB-FUBINACA were evaluated in 2017 by Carlier et al. [10] using liquid chromatography quadrupole time-of-flight mass spectrometry (LC–QTOF/MS). This SC presented the base peak at *m/z* 383.1878 and the characteristic ionic fragments were those produced by the loss of the aminodimethylbutanamide group (*m/z* 253.0772), of the carboxamide group (*m/z* 338.1663), or by the formation of fluorobenzylium ions (*m/z* 109.0448). Two smaller fragments formed by the loss of the groups dimethylbutanamide (*m/z* 270.1037) and amine (*m/z* 366.1612) were also identified. The *m/z* 109, *m/z* 253 and *m/z* 338 fragments of ADB-FUBINACA were also previously observed in 2013 by Uchiyama et al. [9] using the LC-QTOF/MS with a photodiode array (PDA) detector, and in 2014 by Takayama et al. [38] using ultraperformance liquid chromatography with electrospray ionization-tandem mass spectrometry (UPLC/ESI-MS/MS). On the other hand, the analysis of the characteristic fragment ions of AMB-FUBINACA revealed that the amide bond was most susceptible to cleavage, thus forming a fragment ion at *m/z* 253.0772 (C_15_H_10_FN_2_O^+^), and the cleavage between the indazole ring and fluorobenzyl produced the fluorobenzyl ion (C_7_H_6_F^+^) at *m/z* 109.0448 [39]. Additional ions (*m/z* 145, 324, 383) were reported by Kevin et al. [40], using gas chromatography coupled to mass spectrometry (GC-MS).

Information on the stability of SCs after several cycles of freezing/thawing and the long-term stability in serum and/or other biological fluids exposed to different temperatures are necessary to produce ideal pre-analytical conditions and ensure the adequate storage of biological samples in forensic cases where the use of SC is suspected. Presently, there are little data on the stability of SCs in biological samples. In 2017, using liquid chromatography–tandem mass spectrometry (LC-MS/MS), Hess et al. [41] tested the stability of ADB-FUBINACA in human plasma, at a concentration of 1.5 ng/mL. The authors concluded that ADB-FUBINACA did not remain stable in plasma exposed to three freeze/thaw cycles (each cycle: 20 h at −20 °C and 1 h at 4 °C), so unnecessary freezing and thawing of biological samples where this SC is suspected should be avoided. They also found that ADB-FUBINACA remained stable when the samples were stored at −20 °C and 4 °C for 105 days, and at 20 °C for 315 days (maximum storage times in the study at the respective temperatures). Tynon et al. [42] tested the stability of ADB-FUBINACA in human blood. Samples were stored at room temperature (with and without exposure to light), at 4 °C or frozen (−20 °C). ADB-FUBINACA was shown to remain stable for the maximum length of the study (30 days) at all these temperatures.

Kevin et al. [40] assessed the thermal stability of AMB-FUBINACA, and concluded that this substance produces thermolytic degradants when heated above 400 °C, which is the minimum temperature to which SCs are subjected when smoked. Specifically, AMB-FUBINACA lost the methyl ester substituent and the pendant naphthyl moiety, leaving only the amide linked to the indazole substituent. The amide was further dehydrated to nitrile, which was lost at 400 °C. The authors also observed that at this temperature 25 µg/mg cyanide was formed; thus, AMB-FUBINACA smokers are probably exposed to this toxicant. Of note, the specific thermal degradants (1-(4-fluorobenzyl)-1*H*-indazole, 1-(4-fluorobenzyl)-1*H*-indazole-3-carbonitrile, 1-(4-fluorobenzyl)-1*H*-indazole-3-carboxamide, 1-(4-fluorobenzyl)-*N*-isobutyl-1*H*-indazole-3-carboxamide, and methyl-(1-(4-fluorobenzyl)-1*H*-indazole-3-carbonyl)glycinate) might be interesting analytical targets in AMB-FUBINACA smokers, as they will be potentially more abundant than the parent drug.

## 4. Methods for SC Detection

The development of fast and sensitive analytic methods for the detection and identification of potentially dangerous SCs has been a high priority among the scientific community [43,44], as monitoring drug seizures and substance use is essential for public regulatory and law-enforcement agencies, as well as for clinical and forensic institutions [45].

Most analytical tests for the presumptive analysis of cannabis use are based on the detection of the Δ^9^-THC and its main metabolites, i.e., 11-hydroxy-Δ^9^-tetrahydrocannabinol (∆^9^-THC-OH) and 11-*nor*-9-carboxy-Δ^9^-tetrahydrocannabinol (∆^9^-THC-COOH) in urine [46]. Due to their structural and metabolic differences, these screening tests cannot be used to search for SCs (except for the SC class of ∆^9^-THC analogues) in biological samples such as urine or blood, thus hampering their detection [47]. On the other hand, ELISA-based assays used for SC screening are very limited when confronted with the wide range of different SCs, presenting a high risk of false positives/negatives (e.g., the CEDIA^®^ Assay used for AB-FUBINACA presents cross-reactivity with ADB-FUBINACA) [4,48]. Currently, there are no specific presumptive methods developed for the easy and fast detection of ADB-FUBINACA or AMB-FUBINACA.

GC-MS and LC-MS are the methods most frequently used to detect and quantify ADB-FUBINACA and AMB-FUBINACA in biological and/or seized samples [2,3,39,40,49,50,51], as presented in Table 2 and Table 3, respectively. LC-MS is the preferred technique for the analytical determination of these thermally unstable compounds [52,53,54]. GC-MS shows more limitations in the detection and quantification of the metabolites derived from SCs due to their high polarity and low volatility, the derivatization of the target drugs/metabolites thus being required [55] to increase their volatility and thermal stability and to improve their chromatographic properties [56]. The main challenges of GC-MS and LC-MS lie on the expensive costs associated with the operation and maintenance of the required equipment. In addition, these methods are time-consuming and demand skilled labour to perform the analysis [57]; hence, they are mainly applied in confirmatory tests. Still, the detection and/or quantification of ADB-FUBINACA and AMB-FUBINACA is arduous and challenging since there are no standardised protocols for the identification of these substances. In addition, to the best of our knowledge, there are only a few studies carried out to date attempting to elucidate the metabolites of these NPS, which are rapidly and extensively metabolised, resulting in the altered excretion of compounds in urine [5,10,20,29,39,57], thus further hampering the detection targeted at the parent compounds.

Other methods frequently used in the identification and analysis of AMB-FUBINACA include ultraviolet-visible (UV-Vis) spectrophotometry such as Fourier transform infrared spectroscopy (FTIR) in attenuated full reflection mode [29], LC-MS quadrupole time of flight [3], GC-MS coupled with infrared [58], ion chromatography [58], high-performance liquid chromatography (HPLC) coupled with time-of-flight mass spectrometry [58] and nuclear magnetic resonance spectroscopy [40,58]. Surface-enhanced Raman spectroscopy (SERS) was also used by Islam et al. [57] who detected concentrations of this substance as low as 1 nM, confirming the application of SERS as a fast and sensitive analytical tool in the detection of traces of AMB-FUBINACA and of α-pyrrolidinopentiophenone (a synthetic stimulant of the class of cathinones), also being possibly applied to other NPS. The simultaneous identification of these compounds, however, represents a challenge for SERS when they are present as mixtures, and it is necessary to verify if a separation technique, such as thin-layer chromatography, can be coupled to SERS to overcome this obstacle.

## 5. Prevalence and Patterns of Abuse

Like other SCs, the limited information regarding the production and trafficking of ADB-FUBINACA and AMB-FUBINACA is probably due to limitations in the chemical detection of this type of substances. Nevertheless, the detection of these substances in shipments that are confiscated by European country authorities [6] suggests that AMB-FUBINACA and ADB-FUBINACA are predominantly synthesised in chemical companies based in China (where these substances are not legally regulated), being subsequently processed and packaged in the country to which they are shipped. Traditionally, SCs come in the form of a white or sometimes yellowish powder that is dissolved in organic solvents and subsequently sprayed on herbal products, allowing the user to consume it through inhalation of the smoke after combustion, similar to the way herbal cannabis is smoked in cigarettes [3,29]. Recently, AMB-FUBINACA was also identified in liquid form, which facilitates its consumption through electronic cigarettes and micro-seals, possibly reflecting the ease of adapting the formulations of these substances to the users’ needs [66]. While ADB-FUBINACA was detected in samples of products labelled as “Black Mamba”, “VaperFi”, “Freeze”, and “Mojo” [4,5,60,67], its AMB-FUBINACA analogue has been detected in products marketed under the name “AK-47 Carat Gold”, “Train Wreck2” and “Scooby Snax Limited Edition Blueberry Potpourri”, which consist of mixtures of herbs ready to be used in vaporization devices, electronic cigarettes, inhalers, or even orally ingested [29,40,49]. In addition, there are dozens of other street names for unspecific SC preparations, such as “K2”, “K2XXX”, “barely legal”, “iBlaze”, “spice”, “herbal incense”, “Kush”, and “zombie”, that contain one or more unidentified SCs, including AMB-FUBINACA or ADB-FUBINACA [7].

A study carried out in Ankara and nearby Turkish cities investigated the seizures of illicit herbal substances containing SCs, between 2011 and 2015, and concluded that ADB-FUBINACA was the most commonly found SC [59]. In the EU, no further concrete data are available on ADB-FUBINACA or AMB-FUBINACA confiscations, but in 2015, the seizures of SCs corresponded to 77% of the NPS apprehended, from a total of more than 2.5 tons, with 64% being in the form of herbaceous mixtures and 13% as powders [68]. In 2016, although SCs were the NPS with the highest record of seizures, there was a considerable decrease down to 1.5 tons seized, with herbaceous mixtures corresponding to 40% of the total SCs seized and powder mixtures accounting for almost 13% [6]. According to the EMCDDA, 179 SCs were detected in 2017, of which 10 appeared for the first time, with the statistics indicating that the number of SC seizures in Europe was, at that time, 51% of all NPS [69]. Estimates of SC consumption among the European population aged 15 to 34 ranged between 0.1% in the Netherlands and 1.5% in Latvia, in 2018; and 0.3% in Spain and Lithuania and 0.6% in Italy, in 2019. In addition, data from 15 hospitals monitored by the European Drug Emergencies Network Plus, from 2014 to 2017, indicate an increase in emergency room visits related to SC use during this period [69]. According to an EMCDDA report of 2019, Turkey announced a considerable rise in SC-related deaths: from 137 cases in 2015 to 563 in 2017. In fact, SCs were detected in 60% of the total number of drug-related deaths recorded in the country during that period, with the majority of the cases related to young men aged around 20 years [69]. More recently, the 2020 European drug report showed that SCs together with cathinones represent 77% of all seizures notified in 2018 [1].

In the USA, in 2017 and in the first half of 2018, AMB-FUBINACA was the most frequently identified SC in drug seizures by the DEA [70]. On the other hand, ADB-FUBINACA was the third most recurrently identified SC in 2018 [in 71 out of 526 seizures (13%)]; these values decreased to 7% in 2019, probably as a result of its inclusion in the Schedule I category of the Controlled Substances Act [71,72,73].

Despite the varied profiles of SC abuse, since these substances have a reputation of causing psychotropic effects at a relatively low cost, the increasing use of SCs by vulnerable groups such as homeless people and prisoners has recently emerged as a particularly concerning pattern. Adding to the commonly reported adverse effects, the SC prison market has been associated with an increase in aggression and violence, and in some countries this has caused a serious threat to general security in the penitentiary environment [6,7,68].

## 6. Legal Status

AMB-FUBINACA and ADB-FUBINACA are in the process of becoming subjected to international control under the 1971 United Nations Convention on Psychotropic Substances and the 1961 Single Convention on Narcotic Drugs [29]. Accordingly, the EU has already issued a favourable opinion on the inclusion of AMB-FUBINACA, ADB-FUBINACA and other SCs in the tables of the aforementioned conventions [74].

In 2017, the DEA recognised ADB-FUBINACA and AMB-FUBINACA as being significantly dangerous and issued a temporary statement that placed these drugs in the Schedule I category of the Controlled Substances Act to limit the imminent risk to public safety. This resulted in the application of regulatory controls and administrative, civil, and criminal sanctions against anyone who handled or proposed to handle these SCs [71,72,73]. In 2020, after evaluating the clinical and scientific data and considering the recommendations of the US Department of Health and Human Services, the DEA determined that AMB-FUBINACA and ADB-FUBINACA were to be permanently placed in the category of controlled substances [75,76]. These drugs are also banned in Canada, where they are classified as narcotics under the Canadian Drug and Controlled Substances Act, which means that the possession and trafficking of AMB-FUBINACA and ADB-FUBINACA are punishable by law with up to a maximum of five years in prison, and their production or export may be punishable with life imprisonment [77,78]. In 2017, Health Canada issued a warning to Canadians regarding the illegal sale of some SC-containing products at establishments with a legal license to market cannabis and cannabis-derived products in Edmonton [78]. The AMB-FUBINACA regulations are also being reviewed by the New Zealand Ministry of Health.

In Europe, ADB-FUBINACA is monitored by the EMCDDA as an NPS under the Regulation No. 1920/2006 of the European Parliament and Council [79]. ADB-FUBINACA was already detected in 19 Member States and is controlled in at least ten. For example, in Germany, it is covered by the *Anlage II* narcotics law [74]. Although the EMCDDA did not issue an alert or carry out any risk assessment of AMB-FUBINACA, this substance is considered illegal by annex II of the Narcotics Law (Directive EU/2019/369, of 13 December 2018), was under surveillance until 2016 by the German Controlled Substances Act [80], and is prohibited in Sweden by the Swedish National Public Health Authority [29]. In Portugal, the Law no. 58/2020 from August 31 included AMB-FUBINACA and ADB-FUBINACA in the tables of substances attached to Decree-Law n 15/93 of January 22, which regulates the legal status of the misuse of drugs [81,82].

## 7. Subjective and Other Biological Effects

The most common effects elicited by AMB-FUBINACA and/or ADB-FUBINACA in humans, as described either in clinical cases or mentioned by users in drug forums, can be classified into two major groups—psychological and physical effects. Reports from drug forums lack scientific rigor as they consist of subjective observations and consumers are often unsure whether AMB-FUBINACA and/or ADB-FUBINACA were present or mixed with other substances [2,3,23,83,84,85,86].

The acute psychological effects of AMB-FUBINACA and ADB-FUBINACA may be similar but more severe to those reported during an acute cannabis intoxication [3,23,83,84,85,86,87]. These effects are felt 10 to 15 s after administration, peaking between 5 to 20 min, and last about 45 to 60 min. They comprise euphoria, relaxation, feelings of anguish, confusion, anxiety, and fear [3,23,83,84,85,86,87]. Users of online forums frequently mention drowsiness, dizziness, delusions, agitation, headache, verbiage, psychedelic effects, and an altered perception of sounds [3,23,83,84,85,86,88,89], with more susceptible individuals experiencing a distorted perception of time, hallucinations, paranoia, and even the development of psychiatric disorders. Less common but more severe psychological effects have also been reported, including severe psychosis, catatonia, or coma [3,23,84,85].

Most recurrent physical effects include eye flushing (ocular vascularization), tachycardia, chest pain, nausea, vomiting, seizures, myoclonus, and impaired motor performance [3,23,29,83,84,85,86]. In addition, pathologically severe conditions, namely encephalopathies, hypertension, stroke, acute kidney injury, and renal failure, have also been documented [3,7,23,83,84,85,86,90]. Hamilton et al. [49] suggested that the acute myocardial infarction with elevation of the ST segment observed in consumers of AMB-FUBINACA may be an adverse effect transversal to several SCs. The consumption of AMB-FUBINACA was also associated with rhabdomyolysis [91].

## 8. Clinical Toxicology

Since SCs are in general more potent in their action than phytocannabinoids, the effects experienced, even those derived from the use of lower doses, are generally more severe and can even be fatal [3,6,23,84,86]. Moreover, biological effects appear at shallow doses, precipitating the occurrence of toxicity and overdose in inexperienced users [9,10,18]. It is equally concerning that AMB-FUBINACA and ADB-FUBINACA are frequently consumed mixed with other potentially toxic substances, which may widen the range of adverse effects or result in toxicities higher than originally expected for the single drugs. On the other hand, SC products may display “hot spots” resulting from the poor homogenization of their components, which may aggravate the risk of intoxication [7]. Moreover, combustion of AMB-FUBINACA and ADB-FUBINACA involves the thermal transformation of these SCs, leading to the release of highly toxic molecules, including cyanides, toluene, naphthalene, and 1-naphthalamine. The neurological and cardiovascular effects of cyanide may potentiate SC complications [29].

Several clinical cases have shown that acute administration of ADB-FUBINACA and AMB-FUBINACA contributes to serious adverse effects and fatalities [2,3,4,5,60,61,67]. In each case described, the drugs were confirmed analytically by testing the consumed product and/or the consumers’ urine or blood. However, in most cases, other substances, including other SCs, were also present (Table 4).

In 2017, Lam et al. [4] reported a case in Hong Kong involving a healthy 24-year-old male who smoked, using an electronic cigarette, two drops of “VaporFi”, a product whose analysis revealed the presence of AB-FUBINACA and ADB-FUBINACA. About 30 min after inhalation, the patient became drowsy, confused, and agitated, with palpitations and vomiting, and when entering the emergency room, a short period of supraventricular tachycardia appeared, which soon resolved itself. The immunoassay performed in urine to detect drugs of abuse was negative. However, exposure to AB-FUBINACA and ADB-FUBINACA was confirmed analytically by LC-MS/MS, in the blood sample collected, with serum concentrations of 5.6 ng/mL and 15.6 ng/mL, respectively. The patient recovered uneventfully with supportive treatment and was discharged 22 h after admission. Moeller et al. [67] also reported in the same year a case in Oldenburg, Germany, involving a 25-year-old man who had severe left hemiparesis and left hypaesthesia, moderate dysarthria and visual neglect. Magnetic resonance angiography and ultrasound examination revealed an occlusion of the right proximal middle cerebral artery. The patient had smoked 3 g of a product called “Freeze”, on the previous night. This product was later analysed by GC-MS, with ADB-FUBINACA being detected. The patient’s urine was positive for ADB-FUBINACA and MDMB-CHMICA, and the serological tests suggested that the patient had not consumed other psychoactive drugs. The authors believed that the cardiac sympathomimetic effect of the product consumed may have triggered an unnoticed episode of tachyarrhythmia that resulted in a stroke of cardioembolic etiology. Of note, there are other reports that also document strokes of possible cardioembolic origin after consuming products containing SCs [93,94,95]. In the following year, Nacca et al. [61] described a case in New York involving a 38-year-old male who was hospitalised with altered mental status and bradycardia. Later, the patient showed progressive encephalopathy and seizures accompanied by autonomic instability, respiratory failure, type-I second-degree atrioventricular block, hypotension, hypothermia, and hypoglycaemia, so a computed tomography scan was performed, detecting several broken packages in the stomach and rectum. The patient was submitted to a surgery to remove the packages, and during the recovery period presented with generalised and focal seizure activity. During the following week, his mental state progressively returned to normal, being discharged one month after the event, with no neurological sequels recorded. The serum, urine, and package contents analyses by LC-QTOF/MS identified ∆^9^-THC and ADB-FUBINACA, being the ADB-FUBINACA blood concentration of 34 ng/mL (89 nM), the highest documented to date in cases of non-fatal intoxications.

In addition to these cases of non-fatal intoxications, fatalities associated with the use of ADB-FUBINACA have also been described. Shanks et al. [5] reported, in 2016, a case in East Baton Rouge, Louisiana, involving a 41-year-old woman with a history of SC inhalation, in which a serum concentration of 7.3 ng/mL ADB-FUBINACA was determined in the autopsy, the cause of death being certified as coronary artery thrombosis following the drug use. In a case reported in 2019 by Chan et al. [92], a 17-year-old Chinese man passed away after smoking an unknown product in Singapore. Immediately after consumption, he experienced uncontrollable tremors and vomiting. Six hours later, he entered the emergency room, already dead. At the autopsy, samples of peripheral blood (femoral) and urine as well as stomach and biliary contents were obtained and sent for toxicological analysis. ADB-FUBINACA was identified at a concentration of 56 ng/mL (146.5 nM) in blood, the highest documented to date. No alcohol or other drugs were detected, so the cause of death was attributed to the toxicity of ADB-FUBINACA.

In 2016, the authorities of New York City witnessed a massive intoxication of 33 people by AMB-FUBINACA. This episode was dubbed a “zombie outbreak” by the local media due to the appearance of the intoxicated users, who showed symptoms of severe depression of the central nervous system. For example, one of the consumers assisted in the emergency department had a state of marked lethargy, only reactive to tactile stimuli, besides showing guttural moans, marked sweating and slowing of the movements of the upper and lower limbs [3]. In this specific case, the toxicity generated by AMB-FUBINACA was not associated with tachycardia, seizures, cardiotoxicity or renal failure [3]. AMB-FUBINACA and its 5F-ADB counterpart were also responsible for several non-fatal hospitalizations and 34 deaths in Auckland city in New Zealand in 2017, with 40 to 45 suspected deaths related to these substances having recently been investigated [12,17,96,97]. The high number of fatalities in New Zealand was probably due to the higher amounts of AMB-FUBINACA in the preparations sold, containing an average concentration of 59 mg/g, while the samples seized in New York [3] contained an average of 16 mg/g. In this sense, an association can be made between adverse toxic effects and the dose of AMB-FUBINACA [3,29,97].

Although comparison between the toxicities of ADB-FUBINACA and AMB-FUBINACA in humans are precluded by discrepancies in the exposure conditions (dissimilarity of doses and routes of administration, interindividual variability, the co-occurrence of other substances, the time elapsed between the drug administration and the clinical manifestation or the drug quantification in the biological fluids, etc.), studies in animals do not indicate significant differences between these drugs. Accordingly, Gatch et al. [98], in 2019, injected ADB-FUBINACA and AMB-FUBINACA into mice at doses between 0.1 and 1 mg/Kg and 0.1 and 0.5 mg/Kg, respectively, observing an ED_50_ of 0.19 mg/Kg for both SCs. In addition, the depressant effects in locomotor activity, as indicated by the appearance of tremors, were observed 60 to 90 and 30 min after administration of ADB-FUBINACA and AMB-FUBINACA, respectively.

### 8.1. Mechanisms of Toxicity

The exact mechanisms by which SCs, including AMB-FUBINACA and ADB-FUBINACA, produce their wide range of harmful effects, are not fully understood, and to date, there are few preclinical assessments of their acute or chronic toxicological effects [29]. In addition, it is also unknown whether the toxicity of these compounds is caused by the parent compounds (which are rapidly metabolised) or by the action of metabolites and/or thermolytic products [99,100,101]. In this line, ADB-FUBINACA metabolism in humans involves the formation of epoxides, which are highly reactive molecules that have long been identified as biologically harmful, causing toxicity and carcinogenicity [102,103] through the covalent binding to nucleophilic centres in proteins and nucleic acids, altering their functionality [102,103,104].

It spite of the lack of knowledge on the toxicological mechanisms of AMB-FUBINACA and ADB-FUBINACA, it is recognised that the difference between the doses that cause the psychoactive effects and the doses that cause toxic effects is small, and that the subjective effects of cannabinoids sought by consumers are due to the CB1R activation [98]. It is important to note that previous studies in vivo with other SCs corroborate the symptoms reported in clinical cases of ADB-FUBINACA users. Accordingly, Banister et al. [20] evaluated, by biotelemetry performed on rats, the cannabimimetic activities, specifically the change in body temperature and heart rate, induced by AB-FUBINACA (structurally differing from ADB-FUBINACA by lacking one methyl group) and its demethylated derivative AB-PINACA. Doses of 0.1, 0.3, 1, and 3 mg/Kg of each SC were administered intraperitoneally at two day-intervals, to promote the elimination of the compound. The temperature of the rats was evaluated from 1 h before to 6 h after drug administration, at 15-min intervals. The heart rate was evaluated over the same time span, every 30 min. The dose of 0.1 mg/Kg showed no significant cannabimimetic effect. However, the remaining doses triggered hypothermia (a decrease of 2 °C of body temperature, for the doses of 0.3 to 3 mg/Kg of AB-FUBINACA; a decrease of 1.5 °C of body temperature, for doses of 0.3 to 3 mg/Kg of AB-PINACA) and bradycardia (for both SCs there was a decrease of 100 to 150 bpm 1 h after administration of 3 mg/Kg) in the tested mice. Assays performed in the presence of rimonabant and SR144528, two selective antagonists of CB1R and CB2R, respectively, showed that the symptoms described were reversed in the presence of the CB1R antagonist, but not in the presence of the CB2R antagonist.

### 8.2. Treatment

There is no specific treatment described for the cases of acute toxicity by AMB-FUBINACA or ADB-FUBINACA. However, the general treatment of acute poisoning by SCs is often performed through supportive measures, namely by controlling signs and symptoms and fluid therapy to obviate electrolyte disturbances [6,85]. Patients experiencing irritability, agitation, anxiety and seizures, both associated with SC intoxication and withdrawal syndrome, are usually treated with benzodiazepines as the first-line approach. Neuroleptics are also administered to manage psychotic symptoms [105,106].

## 9. Pharmacodynamics

**Figure 2:**
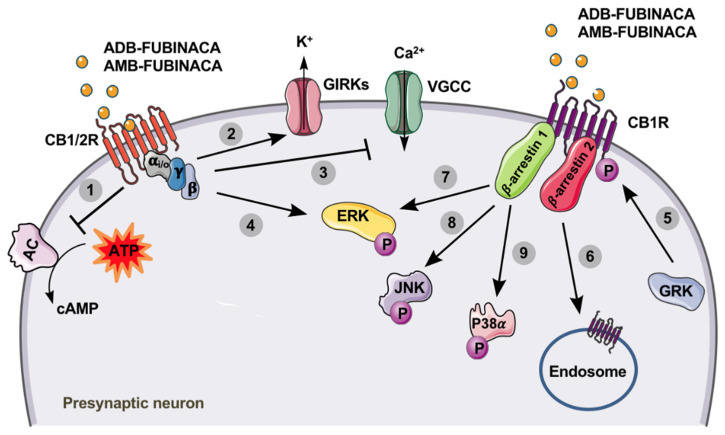
Cannabinoid receptors type 1 (CB1R) and 2 (CB2R) are members of the G protein-coupled receptor (GPCR) family, being associated with the G_i/o_ type. After activation by ADB-FUNIBACA or AMB-FUBINACA (yellow circles), the activity of adenylate cyclase (AC) is inhibited (1), resulting in decreased levels of cyclic adenosine monophosphate (cAMP). Additionally, the opening of G protein-coupled inwardly-rectifying potassium channels (GIRKs) increases K^+^ efflux (2), while the inhibition of voltage-gated calcium channels (VGCC) decreases presynaptic Ca^2+^ influx (3). These events lead to the suppression of neurotransmitter release at excitatory and inhibitory synapses [108]. The binding of AMB-FUBINACA to the CB1R also results in increased extracellular signal-regulated kinase (ERK) activation (phosphorylation), through G_i/o_-protein-mediated signalling (4), which ultimately results in the modulation of cell proliferation, differentiation and survival. After activation of CB1R by ADB-FUNIBACA or AMB-FUBINACA, the phosphorylation of this receptor by G protein-coupled receptor kinases (GRK) can occur (5), making it highly susceptible for the binding of *β*-arrestins. Consequently, receptor desensitization and internalization are stimulated (6), which is *β*-arrestin 2-dependent; and a variety of signalling cascades, mostly mediated by *β*-arrestin 1, are promoted, including ERK (7), c-Jun *N*-terminal kinase (JNK) (8) and P38*α* (9) activation (phosphorylation) [109,110].

ADB-FUBINACA is a potent CB1R agonist, with a binding affinity (Ki, inhibition constant) of 0.36 nM and an EC_50_ value of 0.98 nM for [35S]GTPᵧS (the assay measures the level of G protein activation after occupation of the coupled receptor) [8]. Banister et al. [20] analysed the binding of several SCs to CB1R and CB2R expressed in mouse neuroblastoma AtT20-FlpIN cells, concluding that the efficacy and potency of ADB-FUBINACA were substantially higher than those of ∆^9^-THC, as measured by the opening of internal potassium rectification channels dependent on G protein (GIRKs). Higher GIRK channel functional activity was obtained in CB2R compared to CB1R, with EC_50_ values of 3.5 nM and 1.2 nM, respectively. In a similar in vitro study, Noble et al. [19] assessed the structure-potency relationship of 14 SCs in human embryonic kidney HEK239 cells transfected with CB1R and CB2R using an activation assay of these receptors. ADB-FUBINACA was the most potent SC tested, activating the signalling pathway of β-arrestin 2 (Figure 2), with EC_50_ values of 0.69 nM and 0.59 nM in CB1R and CB2R, respectively; and an Emax about three times higher than that of the SC JWH-018 on CB1R. Of note, the potency of AB-FUBINACA-COOH, which is a metabolite common to AB-FUBINACA and AMB-FUBINACA, was also evaluated. Although the results suggest that the metabolite retains significantly lower pharmacological activity at CB1R and CB2R than the parent drug, concentrations achieved in vivo (up to 636 ng/mL or 1.72 µM) are enough to partially activate the CBRs [3]. This study also concluded that structural differences in SCs result in large differences in the affinity for CBRs (e.g., the chlorine substitution enhanced the potency at CB1R compared with other halogenated analogues), which may be correlated with the disparities found in toxic effects in humans. More recently, Wouters et al. [107] analysed the activity of seven metabolites obtained by hydrolysis of 15 SCs, including ADB-FUBINACA. For this purpose, HEK293T cells were used as a model to determine the activity of the SCs and their metabolites in CB1R. The authors observed that ADB-FUBINACA showed EC_50_ values of 0.82 nM and Emax of 273.6% in CB1R, while its ADB-FUBINACA-COOH metabolite (resulting from terminal moiety hydrolysis) produced a significantly lower activity at the CB1R, compared to the parent compound (EC_50_ 450 nM; Emax 176.6%), indicating that metabolism of ADB-FUBINACA can potentially contribute to reduce the CB1R-mediated pharmacological and/or toxicological response(s). Pharmacological mechanisms described in vitro for ADB-FUBINACA are depicted in Figure 2. To the best of our knowledge, there are currently no published data regarding the in vivo pharmacology of ADB-FUBINACA.

AMB-FUBINACA is also a potent agonist for CBRs, displaying a Ki of 10.04 nM for CB1R ([3H]SR141716A as the reference ligand) and 0.79 nM for CB2R (ligand [3H]CP55,940 as a reference), both expressed on the HEK293 human cell membrane after transfection [17]. In fact, in vitro pharmacological studies estimated that AMB-FUBINACA is about 85 times more potent than ∆^9^-THC and 50 times more potent than JWH-018, another frequently consumed SC [11,20,111]. As observed in tests of affinity to [35S]GTPγS [11,17], AMB-FUBINACA is a full agonist of CB1R (EC_50_ 0.54 nM), but proved to be less potent than the SC of reference, CP55,940 (EC_50_ 0.18 nM). However, in the inhibition of cAMP and stimulation of GIRK (Figure 2), AMB-FUBINACA (EC_50_ 0.63 nM and 2.0 nM, respectively) was shown to be more potent than CP55,940 (EC_50_ 2.1 nM and 42 nM, respectively). AMB-FUBINACA also proved to be a full agonist of CB2R with a binding strength similar to that of CP55,940, with EC_50_ values of 0.13 nM and 0.14 nM, respectively [17,54]. In the GIRK stimulation test, the affinity of AMB-FUBINACA in CB2R was lower than that of CP55,940, with EC_50_ values of 18 nM for AMB-FUBINACA and 4.2 nM for CP55,940 [11,17]. Finlay et al. [87] evaluated the functional selectivity of AMB-FUBINACA for the fundamental pathways of receptor activity, including the cAMP inhibition pathway, activation of the extracellular signal-regulated kinase (ERK), internalization of CB1R, and translocation of ß-arrestin 1 and 2. The results revealed that AMB-FUBINACA is highly effective and potent in the activation of all tested pathways [87]. In view of their high affinity for binding and activating CB1R, AMB-FUBINACA and ADB-FUBINACA have been shown to completely substitute for ∆^9^-THC in male C57/Bl6 mice, trained to discriminate the vehicle from ∆^9^-THC, with an ED_50_ of 0.44±0.14 mg/Kg [17,29]. This discriminatory effect was confirmed in another study, where it was also observed that high concentrations (1 mg/Kg) of AMB-FUBINACA induced seizures [98].

Recently, the chemical structure of SC-linked CB1-Gαi complex (α subunit of the G protein complex) has been disclosed by using electronic cryo-microscopy [28]. The indazole skeleton shared by many SCs, including AMB-FUBINACA and ADB-FUBINACA, was demonstrated to interact with the amino acid residue F200^3.36^, helping to stabilise the connection to the CB1R, and allowing the rotation of the W356^6.48^ of the receptor to form an interaction cavity with the Gαi subunit on the cytoplasmic face of the receptor [28]. Kumar et al. [28] demonstrated that this interaction, called “twin-toggle switch”, is highly efficient for MDMB-FUBINACA, a SC analogue differing only by the addition of a methyl group to the valinate side chain of AMB-FUBINACA. Given its structural similarity, the “twin-toggle switch” interaction is probably common to AMB-FUBINACA, this being the main difference between full agonists such as SCs, and partial agonists like ∆^9^-THC. Due to its structural rigidity, MDMB-FUBINACA locks “toggle switch” residues F200^3.36^/W356^6.48^ in active receptor conformation, triggering a much faster and more efficient activation of the receptor than ∆^9^-THC, which is comparatively more flexible [28]. In contrast, the high efficacy of MDMB-FUBINACA is partly due to its structural rigidity in the characteristic C-shape configuration that stereotypically recognises the CB1R binding site and stabilises the MDMB-FUBINACA, blocking the “selector switch” with residues F200^3.36^/W356^6.48^ in the active conformation. The pathway of interaction with Gαi facilitates the canonical effects of this receptor, and might help to explain why AMB-FUBINACA is a highly effective agonist at the nanomolar range [87].

## 10. Pharmacokinetics

Knowledge on the pharmacokinetics of ADB-FUBINACA and AMB-FUBINACA is essential to document abuse. No data on the distribution of these drugs are available in the literature, but due to their lipophilic nature, these drugs are expected to extensively bind to plasma proteins. Information on the absorption, metabolism and excretion are herein compiled.

### 10.1. Absorption

Based on consumer reports, the main route of administration of ADB-FUBINACA and AMB-FUBINACA is presumably the same as that used for other SCs, i.e., inhalation of smoke after combustion of the SC present on the plant matrix [7], or inhalation of the vapours/steam obtained from liquid or oily preparations of the substance by using vaporisers or electronic cigarettes (e-liquid) [112]. The dose required for the pharmacological effects to occur in humans is still unknown, but this route determines rapid drug absorption, and therefore, an immediate central nervous system exposure to the SCs (with the onset of intense pleasure in only a few seconds or minutes). Nevertheless, as observed for ∆^9^-THC smoking [46], pyrolysis may destroy a variable amount of the SCs, exposing consumers to the degradation products. Although no information on ADB-FUBINACA and AMB-FUBINACA bioavailability is reported, it can be considered that it mainly depends on the specific characteristics of the cigarette and/or combustion, the intensity and duration of the inhalation, and the characteristics of the consumer (e.g., chronic smokers *versus* inexperienced people).

### 10.2. Metabolism and Elimination

ADB-FUBINACA and AMB-FUBINACA are emerging SCs whose metabolic data are also still scarce.

**Figure 3:**
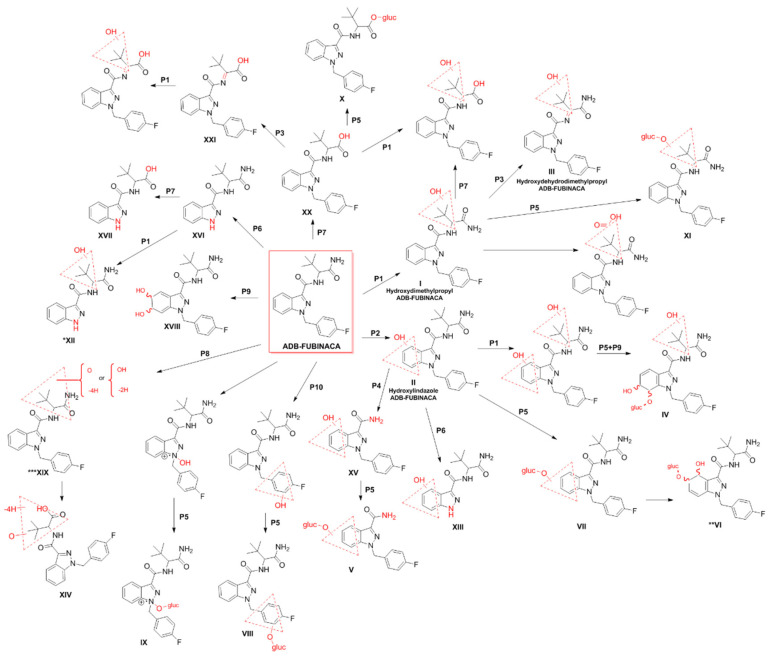
Metabolic pathways of ADB-FUBINACA in humans [38,52]. The main biotransformation pathways include alkyl (P1) or indazole ring hydroxylation (P2), dehydrogenation (P3), secondary amide hydrolysis (P4), and glucuronide conjugation (P5). Other metabolic pathways are *N*-dealkylation (P6), primary amide hydrolysis (P7), carbonylation (P8), epoxidation followed by hydrolysis (P9), and methylene-fluorophenyl hydroxylation (P10). Dashed red triangles represent the location at which the reaction supposedly occurs. *XII is also a metabolite resulting from the *N*-dealkylation of I. **VI is formed by further hydroxylation of ADB-FUBINACA hydroxyindazole-glucuronide on the benzene ring. ***XIX is formed by hydroxylation and a di-dehydrogenation on the amino-dimethylbutanamide moiety.

In 2014, Takayama et al. [38] first attempted to elucidate the in vitro metabolism of ADB-FUBINACA by analysing the metabolites produced by the activity of the cytochrome P450 enzymes after 1 h of incubation with human liver microsomes. Using UPLC/ESI-MS/MS, the authors identified a single metabolite, resulting from oxidation of the *N*-(1-amino-3,3-dimethyl-1-oxobutane) portion (Figure 3; metabolite **I,** which resulted from the methyl hydroxylation at the dimethylpropane chain). Carlier et al. [10] further assessed the metabolic stability of ADB-FUBINACA in the same in vitro model, disclosing a half-life of 39.7 min, with a predicted liver elimination of 9.0 mL/minute/Kg [10]. ADB-FUBINACA was considered an intermediate-clearance drug; therefore, metabolites might be detected in urine several days after consumption. The authors also predicted a significant first-pass hepatic effect when the drug is orally administered, and liver elimination susceptible to alterations in plasma protein binding and hepatic blood flow. It is, however, important to note that, due to its lipophilic nature, ADB-FUBINACA is expected to be a highly protein-bound SC, which could lower the liver elimination and extend the detection window.

Carlier et al. [10] also incubated human hepatocyte suspensions with 10 μM ADB-FUBINACA for up to 3 h, detecting 22 additional metabolites by LC-HRMS (Figure 3). Such differences in the number of metabolites detected in this work and in the study of Takayama et al. [38], might be related to discrepancies in the in vitro models (primary human hepatocyte suspensions *versus* human liver microsomes), the concentrations (10 μM versus 5 μM) and the analytical methods (LC-HRMS *versus* UPLC/ESI-MS/MS) used.

The main metabolic pathways identified in the hepatocyte were alkyl hydroxylation (Figure 3; pathway 1), indazole hydroxylation (Figure 3; pathway 2), dehydrogenation on the aminodimethylbutanamide portion (Figure 3; pathway 3), and hydrolysis of the amide group (Figure 3; pathway 4) with subsequent conjugation with glucuronide (Figure 3; pathway 5). In fact, the presence of multiple metabolites resulting from glucuronidation (Figure 3; Metabolites IV, V, VI, VII, VIII, IX, X, XI) anticipate the need for hydrolysis of biological matrices previous to the extraction to concentrate the metabolites, further facilitating their detection.

Of note, metabolite XV (Figure 3), which is formed by ADB-FUBINACA dimethylbutanamide cleavage, was present at low amounts after an 1 h of incubation but was not detectable after 3 h. Both metabolites XV and V (Figure 3) are also products of AB-FUBINACA metabolism and can hypothetically be formed by metabolism of SCs that display the same indazole-dimethylbutanamide structure. Similarly, other metabolites (e.g., XVI and XVII) may be theoretically formed by *N*-dealkylation of several SCs that share the same (4-fluorobenzyl)indazole structure. As such, based on their specificity as ADB-FUBINACA metabolites and the intensity of the mass spectrometry signals, the authors concluded that ADB-FUBINACA hydroxy-alkyl (Figure 3; Metabolite I), ADB-FUBINACA hydroxydehydroalkyl (Figure 3; Metabolite III, which resulted from dehydrogenation of the aminodimethylbutanamide portion) and ADB-FUBINACA hydroxylindazole (Figure 3; Metabolite II) have the potential to be used as exposure biomarkers. However, the need for confirmation of the above results with in vivo experiments or authentic urine specimens following ADB-FUBINACA intake was highlighted to allow reliable extrapolation of these pharmacokinetic findings to the investigation of clinical and forensic cases. With this purpose, Kavanagh et al. [52] evaluated the metabolites of ADB-FUBINACA in blood and urine collected from patients admitted to the hospital emergency room due to suspected drug intoxication, or from forensic *post-mortem* investigations. In this study, 38 metabolites were identified using LC-QTOF-MS, including metabolites I, II and II (Figure 3) in large quantities. In addition, metabolites XI, XII, XIII and XIV (Figure 3) were detected for the first time [52]. More recently, Kovács et al. [62] reported five metabolites of ADB-FUBINACA and the parent compound in the *post-mortem* blood collected from a 23-year-old regular drug user who died a few hours after the consumption of *N*-ethylhexedrone and ADB-FUBINACA. The autopsy revealed the presence of the ADB-FUBUNACA metabolites resulting from the dihydrodiol formation through epoxidation of the benzene moiety of the indazole ring, followed by hydrolysis of the newly formed epoxide (metabolite XVIII, Figure 3), aliphatic mono-hydroxylation (metabolite I, Figure 3), carbonylation (metabolite XIX, Figure 3), amide hydrolysis (metabolite XX, Figure 3), and amide hydrolysis followed by dehydrogenation (metabolite XXI, Figure 3). ADB-FUBINACA was not, however, considered the cause of death due to the low blood concentration (0.08 μg/L) achieved.

**Figure 4:**
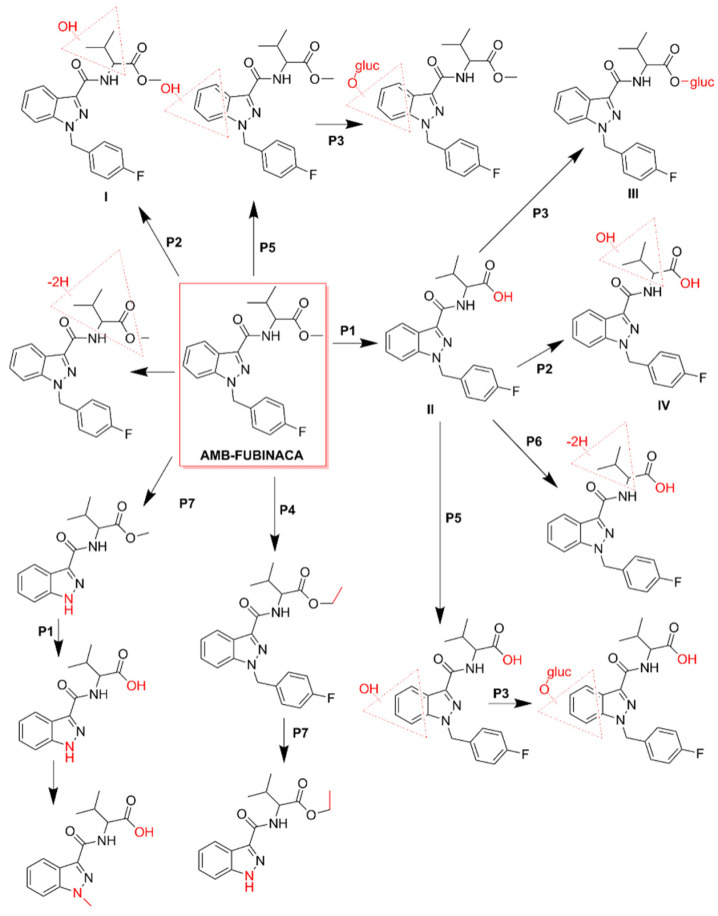
Metabolic pathways of AMB-FUBINACA [45]. The main biotransformation pathways include ester hydrolysis (P1), hydroxylation (P2), and glucuronide conjugation (P3). Methylation (P4), hydroxylation of the indazole ring (P5), dehydrogenation (P6), and *N*-dealkylation (P7) are also displayed. Dashed red triangles represent the location at which the reaction supposedly occurs.

Despite the scarcity of toxicokinetic data available for AMB-FUBINACA, particularly in humans, there are some recent in vitro studies [39,45] suggesting that metabolism is extremely fast, with the demethylation of the parent compound occurring in hepatocytes in just a few minutes (Figure 4; pathway 1). Metabolization is practically complete within 60 min, with only 0.5% of parent drug present at the end of that period [45]. Therefore, from a pharmacological and toxicological perspective, it has been assumed that AMB-FUBINACA metabolites may have a greater relevance for the observed effects than the parent drug itself, although peak effects also occur almost instantly when the SC is smoked [87,113].

The metabolism of AMB-FUBINACA in human liver microsomes resulted in 16 metabolites, being the main phase I metabolic pathways, the ester hydrolysis (Figure 4; pathway 1), hydroxylation (Figure 4; pathway 2) and methylation (Figure 4; pathway 4) [39]. Glucuronidation has been identified as the main phase II metabolic pathway (Figure 4; pathway 3). The most important metabolites result from the hydroxylation of the vanilloid side chain (Figure 4; metabolite I), from the hydrolysis of the terminal ester (Figure 4; metabolites II) followed by the respective conjugation with acid glucuronic (Figure 4; metabolite III). These metabolites can be used as potential biomarkers of exposure in cases of intoxication by this SC [39].

It is worth highlighting that, in the analyses performed on the blood and urine of the patients who received medical care during the referred set of AMB-FUBINACA-related intoxications that occurred in New York in 2016, none of the samples contained the parent compound [3]. These results corroborated several in vitro data previously obtained, supporting the rapid metabolization of SCs after use [45]. Hydrolysis of AMB-FUBINACA occurred rapidly and the de-esterified acid metabolite of AMB-FUBINACA, i.e., 2-(1-(4-fluorobenzyl)-1*H*-indazole-3-carboxamide)-3-methylbutanoic acid (Figure 4; metabolite II), was detected in all patients [3].

## 11. Conclusions

In the last decade, there has been an increase in the consumption of various SCs worldwide. AMB-FUBINACA and ADB-FUBINACA have been the focus of interest by toxicologists, legislators, and health professionals, as their use put the health of many citizens at risk, mainly young adults.

Like most SCs, ADB-FUBINACA and AMB-FUBINACA are highly lipophilic and undergo rapid and extensive metabolism in the human body, making the detection of the parent compounds in biological samples from intoxicated individuals particularly challenging. Defining the best biomarkers of exposure thus urges the assessment of the metabolic profiles of such drugs in the urine and/or blood samples of abusers [114,115]. However, the availability of these samples is limited since the caseworks that could mostly contribute to this investigation are restricted to situations of medical emergency or forensic cases. Alternatively, in vitro models of liver microsomes or human hepatocytes have been used, but these models also have limitations in simulating the complexity of a living organism [116]. For a better understanding of the reported metabolites and the overall kinetics of ADB-FUBINACA and AMB-FUBINACA, new developments are expected in their investigation, in particular on their absorption, distribution, metabolization, and elimination [29].

Despite presenting well-established adverse effects, the cellular and physiological underlying mechanisms are still unknown, and the molecular pathways of toxicity involved in fatal cases are unclear. Identification of an intoxication by these substances is not possible only based on clinical signs as (i) the symptoms overlap with those induced by various drugs, (ii) the use of multiple drugs is frequent in intoxicated patients, and (iii) incorrect information (e.g., regarding the SC used) is often provided by such patients. In addition, there are still no fast and easy-to-operate analytical tests to detect and identify these substances in an acute situation, as the methods available require sophisticated equipment that is not always available at the hospital units that host urgent intoxication cases [117].

More research is also needed to study which molecular pharmacological mechanisms are responsible for systemic and/or local toxicity [87], since knowledge about the biological effects of these SCs is based essentially on case reports. However, a better understanding of their toxicity profiles in vivo and the adequacy of therapy to treat intoxications by these drugs require further investigation, namely in the different target organs. It is also crucial to develop and validate a new set of analytical tools aimed at detecting the metabolites produced in the human body, helping to expand the detection when intoxications occur. More pharmacokinetic and pharmacodynamic studies are also required, as well as analytical monitoring of clinical/forensic cases to confirm the scarce information available, both in the toxicokinetic and toxicodynamic aspects. Addressing all these issues would thus make it be possible to confirm the best exposure biomarkers and improve the methods of analysis to be applied in clinical emergencies and forensic cases involving these substances. Moreover, the gaps in the knowledge regarding the mechanisms of action, the metabolites produced, and the great diversity of effects caused by ADB-FUBINACA and AMB-FUBINACA hamper the creation of an assistance protocol or the discovery of new therapeutic solutions that may help health professionals cope with SC intoxications.

It is expected that this review may thus help the various stakeholders involved in the response to the intoxications caused by ADB-FUBINACA and AMB-FUBINACA in the development of new methods of monitoring and treatment in the clinical scope, as well as increasing the potential of clinical and forensic research that help to combat the scourge of these new synthetic drugs.

## Figures and Tables

**Table 1:** Physicochemical properties of ADB-FUBINACA and AMB-FUBINACA [12,20,29,35,36,37].

	ADB-FUBINACA	AMB-FUBINACA
Chemical Formula	C_21_H_23_FN_4_O_2_	C_21_H_22_FN_3_O_3_
CAS (Chemical Abstract Service) registration number	1445583-51-6	1971007-92-7
Name in the International Union of Pure and Applied Chemistry (IUPAC)	(S)-N-(1-Amino-3,3-dimethyl-1-oxobutane-2-yl)-1-(4-fluorobenzyl)-1H-indazole-3-carboxamide	Methyl (S)-2-[1-(4-fluorobenzyl)-1H-indazole-3-carboxamido]-3-methylbutanoate
Other Designations		FUB-AMB, FUB-MMB and MMB-FUBINACA
Molar Mass	382.4 g/mol	383.4 g/mol
Fusion Point	135–137 °C	Unknown
Solubility	Soluble in dimethyl sulfoxide and ethanol	Soluble in dichloromethane, ethanol and methanol. Low solubility in water
Physical Appearance	Crystalline solid; White powder	Crystalline solid; White to yellowish powder; Slightly sweetish to the taste, with a sweet, somewhat pleasant aroma

**Table 2:** Analytical techniques for the identification and quantification of ADB-FUBINACA.

Sample	Other Substance(s) Analysed	Sample Preparation/Extraction	Method for Analysis	Method Validation Parameters	Results From the Study	Reference (Year)
Illicit herbal-type products sold on the internet between 2012 and 2013	Opioid AH-7921, and other 8 SCs	10 mg of the herbal matrix crushed to powder were extracted with 1 mL methanol under ultrasonication for 10 min. Following centrifugation (5 min, 3000 rpm), the supernatant was passed through a centrifugal filter	LC-QTOF/MS with a photodiode array detector	*n.d.*	ADB-FUBINACA was identified for the first time in illicit products (no quantification)	[9] (2013)
Standards from the *National Institutes of Health Sciences (Tokyo, Japan)* and their metabolites obtained from incubation with human liver microsomes	AB-FUBINACA; AB-PINACA; QUPIC; 5F-QUPIC; α-PVT; and the metabolites produced in vitro	5 mM ADB-FUBINACA (in DMSO) was diluted 1000× in human liver microsome reaction mixture and incubated for 60 min at 37 °C. An equal volume of acetonitrile was added, and the solution centrifuged (10 min, 26,000 *g*). A total of 1 mL of the upper layer was diluted with water–acetonitrile containing 0.1% formic acid, and dried under reduced pressure. The residue was dissolved in 100 μL of 0.1% formic acid	UPLC/ESI-MS/MS	*n.d.*	The ADB-FUBINACA metabolite resulting from methyl hydroxylation at the dimethylpropane chain was disclosed for the first time	[38] (2014)
Samples obtained from laboratory synthesis through *L*-*tert*-leucinamide	AB-FUBINACA; AB-PINACA; ADB-PINACA; 5F-AB-PINACA; 5F-ADB-PINACA; ADBICA; 5F-ADBICA	-	LRMS-ESI; HPLC; LC-MS	*n.d.*	Pharmacodynamic parameters of ADB-FUBINACA were elucidated: EC_50_ 1.2 nM at CB1R and EC_50_ 3.5 nM at CB2R	[20] (2015)
Human *post-mortem* blood from a fatal poisoning	∆^9^-THC; ∆^9^-THC-COOH	Blood specimen was collected from the inferior vena cava in a 60 mL polypropylene bottle, added of sodium fluoride and potassium oxalate, and a 500 μL aliquot extracted at pH 10.2 into hexane–ethyl acetate (98:2). The organic supernatant was evaporated to dryness under nitrogen, and the residue was reconstituted in 50% acetonitrile	LC–MS/MS	**Internal standard:** JWH-122-d9; **LOQ:** 0.2 ng/mL; **LOD:** 0.1 ng/mL; **Accuracy:** at 1.5 ng/mL, *intrarun* 84.6–106.2% and *interrun* 93.7%; at 6 ng/mL, *intrarun* 97.3–111.2% and *interrun* 101.9%; **Precision:** at 1.5 ng/mL, *Intrarun* 3.3–6.7% and *interrun* 11.4%; at 6 ng/mL, *intrarun* 4.8–6.3% and *interrun* 8.6%; **Linearity:** 0.2–10 ng/mL	ADB-FUBINACA: 7.3 ng/mL; ∆^9^-THC: 1.1 ng/mL; ∆^9^-THC-COOH: 4.7 ng/mL	[5] (2016)
Herbaceous samples seized by police from users or dealers, in Turkey between 2011 and 2015	Other 28 SCs	10 mg of each herbal mixture were extracted for 20 min in 1 mL of chloroform under sonication. Subsequently, 10 μL of the extract was evaporated to dryness and dissolved in 200 μL of methanol before injection	GC-MS	*n.d.*	Identification with no quantification. ADB-FUBINACA was the substance most identified (27.11%)	[59] (2017)
DEA Reference Material Collection	-	**NMR**: Dilution of the analyte to 7 mg/mL in CDCl_3_ containing TMS for reference at 0 ppm; **GC-MS**: Analyte dilution of 4 mg/mL CHCl_3_	NMR; FTIR-ATR; GC-MS	**Internal standard:** Dimethylsulfone (NMR)	-	[13] (2017)
Human plasma from real cases	83 SCs	1 mL of plasma was fortified with 100 μL of 50 ng/mL of each internal standard and 500 μL of a carbonate buffer (pH 10). The aqueous phase was extracted with 4 mL of a mixture of *n*-hexane and ethyl acetate (99:1, *v*/*v*). After vortex mixing (1 min) and centrifugation (10 min, 4000 rpm), the organic phase was evaporated under nitrogen. The residue was dissolved in 100 μL of methanol: water: isopropanol (50:35:15, *v*/*v*)	LC–MS/MS	**Internal standards:** JWH-073-d7; JWH-200-d5; MAM-2201-*N*-(5-chloropentyl)analogue-d5	ADB-FUBINACA remains stable when stored at −20 °C and 4 °C for 105 days; ADB-FUBINACA remains stable when stored at 20 °C for 315 days	[41] (2017)
*Cayman Chemical* standards and their metabolites obtained from incubation with human liver microsomes or human hepatocytes	-	**Microssomes:** 100 μM ADB-FUBINACA (in methanol) was diluted 100× in reaction mixture and incubated for 0, 3, 8, 13, 20, 45, and 60 min at 37 °C. Samples were then collected and added with an equal volume of cold acetonitrile. The samples were stored at −80 °C until analysis. The samples were thawed and diluted 100× with mobile phase before injection. **Hepatocytes:** ADB-FUBINACA (methanol) was diluted in hepatocyte suspension to a final 10 μM concentration, and incubated at 37 °C for 0, 1, and 3 h. Reaction was quenched with an equal volume of ice-cold acetonitrile and samples stored at −80 °C until analysis. After thawing, cells were centrifuged (5000 *g* for 10 min, at 4 °C) to remove cell debris and supernatants diluted 5× with 0.1% formic acid in water (mobile phase) before injection. **Note:** Samples were not extracted before injection to increase detection of potential metabolites. However, matrix suppression might impede detection of metabolites with low signal intensity	LC–QTOF/MS	*n.d.*	ADB-FUBINACA hydroxy-alkyl, ADB-FUBINACA hydroxydehydroalkyl and ADB-FUBINACA hydroxylindazole were recommended as biomarkers of exposure	[10] (2017)
1142 blood samples from forensic investigations, including *post-mortem* examinations and driving impairment cases, between March and September 2015	34 SCs	500 μL of whole blood was added of 0.1 ng/μL internal standard and 500 μL 1.0 M TRIS HCl, pH 10.2. Then, separate extractions were performed to optimise recovery of two classes of SCs: **Arylindole compounds:** Tubes were vortexed and extracted with 3 mL of 99% hexane/1% ethyl acetate for approximately 15 min, under agitation. Following centrifugation (10 min; 3500 rpm), the organic layer was evaporated to dryness at 30 °C under a gentle stream of nitrogen. Residue was reconstituted by adding 200 μL of methanol with 1% formic acid **Aminocarbonyl/carboxamide compounds:** Tubes were vortexed and extracted with 3 mL of methyl t-butyl ether for approximately 15 min, under agitation. Following centrifugation (10 min; 3500 rpm), the organic layer was evaporated to dryness at 30 °C under a gentle stream of nitrogen. Residue was reconstituted by adding 200 μL of 50:50 mixture of water with 0.1% formic acid: methanol with 0.1% formic acid	LC–MS/MS	**Internal standard:** AB-FUBINACA-d4; **LOD:** 1.0 ng/mL; **Recovery:** 110 ± 0.268%	ADB-FUBINACA was detected in 34 (2.3%) samples	[42] (2017)
	AB-FUBINACA	0.5 mL of the sample was added to a Toxi Tube-A extraction tube and shaken for 30 min and centrifuged (5 min, 2500 *g*). 1.25 mL of the top phase was dried under compressed air and reconstituted in 0.8 mL of 30% acetonitrile. A 10 µL aliquot was injected	HPLC-DAD; LC-MS/MS; GC-MS; IT-TOF/MS	**Internal standards:** 5F-AB-PINACA, pinezapam, AB-FUBINACA and ADB-FUBINACA	ADB-FUBINACA: 15.6 ng/mL; AB-FUBINACA: 5.6 ng/mL	[4] (2017)
Human blood and urine from eight real cases of “Black Mamba” use prospectively captured through the Colorado site of the Psychoactive Surveillance Consortium and Analysis Network	-	Blood and/or urine samples were collected at the time of presentation. Any drug or paraphilia found with the patient was confiscated and tested. Samples were stored on ice for less than 12 h. Plasma and urine were then frozen at −80 °C, previous to shipment on dry ice to the reference laboratory at the University of California, San Francisco. No further data are available on sample preparation	LC-QTOF/MS	**LOQ:** 31.25 ng/mL	Only five patients had SCs found in blood or urine; three patients tested positive for ADB-FUBINACA (<31.25 ng/mL)	[60] (2018)
Human blood from an ADB-FUBINACA body packer (non-fatal poisoning)	Cannabis and AB-FUBINACA	-	LC-QTOF/MS	Routine validated method (NMS Labs, Willow Grove, PA)	34 ng/mL ADB-FUBINACA	[61] (2018)
Human blood from a fatal poisoning	*N*-ethylhexedrone	To 1 mL blood sample, 30 μL of 5% ammonia solution and 2 mL ethyl acetate were added and mixed by vortex for 1 min. 1.5 mL of the upper layer were evaporated to dryness at 50 °C. The residue was dissolved in 200 μL 50:50, 0.1% formic acid: 0.1% formic acid in acetonitrile, and centrifuged at 7900 rpm for 10 min. 20 μL of the supernatant were analysed	LC–MS/MS	**LOQ:** 10 ng/mL; **LDO:** 0.01 ng/mL; **Recovery:** 98.9% at 1.5 ng/mL; **Precision:** *Intraday:* 9.23% at 0.1 ng/mL; 3.98% at 1.5 ng/mL; 4.75% at 6 ng/mL; *Interday:* 3.60% at 0.1 ng/mL; 1.86% at 1.5 ng/mL; 4.69% at 6 ng/mL; **Linearity:** 0.01–10 ng/mL; r^2^: 0.9972	ADB-FUBINACA: 0.08 ng/mL; *N*-ethylhexedrone: 285 ng/mL	[62] (2019)
Seized samples (tablets, herbs, powders of different types and seals) in Turkey between 2016 and 2017	79 SCs; 6 cathinones; 3 tryptamines; 2 phenethylamines	**Powders and crystals:** dissolved in methanol at 1 mg/mL and further 100× diluted prior to injection. **Herbal samples:** 10 mg were extracted for 20 min in 1 mL of chloroform under sonication. Then, 10 μL of the extract were evaporated to dryness and residue was dissolved in 200 μL of methanol, prior to injection	GC-MS (identification only); LC–MS/MS (in some samples)	*n.d.*	ADB-FUBINACA was the third most identified NPS in the narcotic samples (8.95%)	[63] (2019)
434 seized samples from the Narcotics and Psychotropic Laboratory; 70 human urine samples from non-fatal cases from the Toxicology Laboratory; 6 post-mortem urine samples from the Forensic Medicine Department, in Kuwait in 2018	More than 15 SCs; 3 synthetic cathinones	**Seized samples:** approximately 500 mg of the dried leaves or powder were dissolved in 1 mL of methanol and centrifuged for 10 min at 1253 *g* at 21 °C. The supernatant was used; **Urine:** glucuronide conjugates were hydrolysed by adding 2 mL of 100 mM acetate buffer (pH 5.0) and 50 mL of β-glucuronidase to each mL of urine. The samples were vortexed for 30 s, heated to 65 °C for 1 to 2 h, and allowed to cool previous to solid-phase extraction	LC–MS/MS; GC–MS	*n.d.*	The majority of SCs were indazole-3-carboxamides, such as ADB-FUBINACA and AMB-FUBINACA. The most common SCs were 5F-ADB, AMB-FUBINACA, and 5Cl-AKB-48. Various mixtures of 2, 3, or 4 types of SCs were identified. The most common mixture was AMB-FUBINACA with 5F-ADB. These drugs were mixed, either together or individually, with methamphetamine, tramadol, heroin, ∆^9^-THC, and ketamine. SCs were associated with six reported deaths	[64] (2019)
Human blood from real cases	29 SCs; 4 amphetamines; ∆^9^-THC; ∆^9^-THC-COOH	To 200 μL of sample, 20 μL of internal standard was added for a final concentration of 5 ng/mL. After the addition of 200 μL of 100 mM sodium acetate buffer (pH 5.0), 300 μL of the conditioned blood was loaded onto a supported-liquid-extraction cartridge. Analytes were eluted with 700 μL methyl *terc*-butyl ether (×2). 20 μL of 0.1 M methanolic HCl was then added to elute SCs, and all extracts were dried at 30 °C under nitrogen flow. Residues were reconstituted in 80 μL of 50:50 (*v*/*v*) water: methanol and vortexed prior to centrifugation (3000 rpm, 5 min)	LC–MS/MS	**Internal standards:** JWH-018; N5HP-d5; **LOQ:** 1 ng/mL; **LOD:** 0.1 to 6.0 ng/mL; **Accuracy:** 5.8% at 1 ng/mL; 19.1% at 5 ng/mL; **Precision:** 10.5% at 1 ng/mL; 9.6% at 5 ng/mL; **Linearity:** 1–6 ng/mL; r^2^: 0.999	The validated method allowed for the simultaneous confirmation of 29 SCs and metabolites, 4 amphetamines, and 2 phytocannabinoids in human whole blood. The five most commonly detected SCs in toxicological samples in New Zealand in 2018 were AMB-FUBINACA and/or its acid metabolite, 5F-ADB and/or its acid metabolite, ADB-FUBINACA, 5F-MDMB-PICA acid metabolite, and MDMB-FUBINACA acid metabolite	[65] (2020)

∆^9^-THC: *trans*-∆^9^-Tetrahydrocannabinol; ∆^9^-THC-COOH: 11-*nor*-9-Carboxy-∆^9^-tetrahydrocannabinol; CB1R: Type-1 cannabinoid receptor; CB2R: Type-2 cannabinoid receptor; CDCl_3_: Deuterated chloroform; DEA: Drug Enforcement Administration; DMSO: Dimethyl sulfoxide; FTIR-ATR: Fourier-transform infrared spectroscopy-attenuated total reflection; GC-MS: Gas chromatography–mass spectrometry; CHCl_3_: Chloroform; HPLC: High-performance liquid chromatography; HPLC-DAD: High-performance liquid chromatography with photodiode array detection; IT-TOF/MS: Ion trap time-of-flight mass spectrometry; LC-MS: Liquid chromatography–mass spectrometry; LC-MS/MS: Liquid chromatography–tandem mass spectrometry; LC-QTOF/MS: Liquid chromatography quadrupole time-of-flight mass spectrometry; LOD: Limit of detection; LOQ: Limit of quantification; LRMS-ESI: Low resolution mass spectrometry-electrospray ionization; *n.d.*: No data (method not validated); NMR: Nuclear magnetic resonance; NPS: New psychoactive substances; SC: Synthetic cannabinoid; TMS: Tetramethysilane; UPLC/ESI-MS/MS: Ultraperformance liquid chromatography with electrospray ionization–tandem mass spectrometry.

**Table 3:** Analytical techniques for the identification and quantification of AMB-FUBINACA.

Sample	Other Substance(s) Analysed	Sample Preparation/Extraction	Method for Analysis	Method Validation Parameters	Results From the Study	Reference (Year)
Serum, whole blood, and urine samples from 8 patients among the 18 who were transported to local hospitals; and a sample of the herbal “incense” product “AK-47 24 Karat Gold”, which was implicated in the so-called “Zombie” Outbreak in New York, 2016	De-esterified acid metabolite of AMB-FUBINACA	-	LC–QTOF/MS	**Internal standard:** AMB-FUBINACA	AMB-FUBINACA was identified in AK-47 24 Karat Gold at 16.0 ± 3.9 mg/g. The de-esterified acid metabolite was found in the serum or whole blood of all eight patients, with concentrations ranging from 77 to 636 ng/mL in serum; and in urine of one patient at 165 ng/mL	[3] (2017)
*Cayman Chemical* drug standards	α-PVP and other NPS	2 µL of 0.1 mM AMB-FUBINACA were mixed with 4 µL of silver nanoparticles and 2 µL of MgCl_2_	SERS	**LOD:** 1 nM	Identification of these drugs in a combination pose a challenge for SERS, however this technique is very useful for detecting individual drugs	[57] (2018)
Samples from human liver microsomes in vitro and zebrafish models in vivo	Metabolites of AMB-FUBINACA	**Human liver microsomes:** AMB-FUBINACA at 5 mM (in methanol) was diluted 200× in microsome suspension and incubated for 1 h at 37 °C. Then, uridine diphosphate glucuronic acid trisodium salt was added and incubated for another half an hour. To terminate the reaction 200 µL of acetonitrile was added, followed by centrifugation (13,000 *g*; for 10 min). 100 µL of the supernatant was used after membrane filtering for analysis **Zebrafish** (6–10 months; 0.8–1.2 g): After exposure to 0.1, 0.5, and 1 µg/mL of AMB-FUBINACA (24 °C) for 24 h, zebrafish were removed, cleaned with water and euthanised. The zebrafish were homogenised with a ball mill, and the samples were loaded onto a SPE-Pak@Vac PSA extraction column, which had been conditioned with 1 mL of methanol and 1 mL of water. The column was washed with 1 mL of acetonitrile. The eluent was dried by evaporation at 60 °C under a stream of nitrogen. The residue was reconstituted in 100 µL of flow phase composed of acetonitrile, and 10 µL of the reconstituted solution was injected for analysis	HPLC	*n.d.*	The precision, simplicity and efficiency of the technique proved advantages for the identification of 17 metabolites, making it a useful tool for the detection of polar metabolites, in clinical and forensic contexts	[39] (2019)
*Cayman Chemical* standards	CUMYL-PICA, 5F-CUMYL-PICA, MDMB-FUBINACA, NNEI and MN-18	Each SC was dissolved in acetonitrile at 0.5 mg/mL, and 16 µL were added to a quartz capillary tube loaded into the thermolysis autosampler that passed the individual samples to the thermolysis probe equilibrated at 50 °C, which was then rapidly heated (20 °C/second) to the desired temperature. The samples were heated sequentially to 200, 400, 600, and 800 °C	GC-MS; LC-MS/MS	*n.d.*	SCs heated above 400 °C produce thermolytic, potentially toxic degradants, such as naphthalene, 1-naphthylamine, cyanide and toluene	[40] (2019)
Human blood from real cases	29 SCs; 4 amphetamines; ∆^9^-THC; ∆^9^-THC-COOH	To 200 μL of sample, 20 μL of internal standard was added for a final concentration of 5 ng/mL. After the addition of 200 μL of 100 mM sodium acetate buffer (pH 5.0), 300 μL of the conditioned blood was loaded onto a supported-liquid-extraction cartridge. Analytes were eluted with 700 μL methyl *terc*-butyl ether (× 2). 20 μL of 0.1 M methanolic HCl was then added to elute SCs, and all extracts were dried at 30 °C under nitrogen flow. Residues were reconstituted in 80 μL of 50:50 (*v*/*v*) water: methanol and vortexed prior to centrifugation (3000 rpm, 5 min)	LC–MS/MS	**Internal standards:** JWH-018; N5HP-d5; **LOQ:** 1 ng/mL; **LOD:** 0.1 to 6.0 ng/mL; **Accuracy:** 5.8% at 1 ng/mL; 19.1% at 5 ng/mL; **Precision:** 10.5% at 1 ng/mL; 9.6% at 5 ng/mL; **Linearity:** r^2^: 0.999 with a confirmation ranged between 1 to 6 ng/mL	The validated method allowed for the simultaneous confirmation of 29 SCs and metabolites, 4 amphetamines, and 2 phytocannabinoids in human whole blood. The five most commonly detected SCs in toxicological samples in New Zealand in 2018 were AMB-FUBINACA and/or its acid metabolite, 5F-ADB and/or its acid metabolite, ADB-FUBINACA, 5F-MDMB-PICA acid metabolite, and MDMB-FUBINACA acid metabolite	[65] (2020)

**α-PVP:** α-Pyrrolidinopentiophenone; **∆^9^-THC:** *trans*-∆^9^-Tetrahydrocannabinol; **∆^9^-THC-COOH:** 11-*nor*-9-Carboxy-∆^9^-tetrahydrocannabinol; **GC-MS:** Gas chromatography–mass spectrometry; **HPLC:** High-performance liquid chromatography; **LC-MS/MS:** Liquid chromatography–tandem mass spectrometer; **LOD:** Limit of detection; **LOQ:** Limit of quantification; **n.d.:** No data (method not validated); **SC:** Synthetic cannabinoid; **SERS:** Surface-enhanced Raman spectroscopy.

**Table 4:** Reported intoxications for ADB-FUBINACA and AMB-FUBINACA.

Substance	Type and Circumstances of the Intoxication	Matrix for Analytical Confirmation	Concentration	Other Detected Substances	Clinical Observations	Reference (Year)
ADB-FUBINACA (“Mojo”)	Fatal; In 2015, shortly after smoking an SC product, a 41-year-old female became violent and aggressive with her family. She was physically restrained by her children and eventually became unresponsive. She was declared dead by the emergency personnel a short time thereafter	Blood	7.3 ng/mL (19.1 nM)	∆^9^-THC: 1.1 ng/mL; ∆^9^-THC-COOH: 4.7 ng/mL	Remarkable findings at autopsy included pulmonary oedema, vascular congestion and thrombotic occlusion of the lumen of the left anterior descending coronary artery by haemorrhagic disruption of coronary arterial plaque, as well as ischemia of the anterior left ventricular myocardium	[5] (2016)
AMB-FUBINACA (“AK-47 24 Karat Gold”)	Non-fatal; On July 12, 2016, a mass intoxication of 33 persons in a New York City neighbourhood, in an event described in the popular press as a “zombie” outbreak. From the 18 patients transported to local hospitals, samples from eight were analysed, revealing the presence of a metabolite of AMB-FUBINACA. The herbal “incense” product implicated in the outbreak was also analysed revealing the presence of AMB-FUBINACA	Blood; Urine	**AMB-FUBINACA de-esterified acid metabolite:** 77 to 636 ng/mL (202.37 to 1671.5 nM) in blood; 165 ng/mL (433.7 nM) in urine; no parent compound detected	-	Strong CNS depressant effects that would account for the “zombie-like” behaviour of the users	[3] (2017)
ADB-FUBINACA	Non-fatal; A 24-year-old man considered healthy was taken to the medical emergency room due to acute confusion, agitation, visual hallucinations, and palpitations. 30 min before arrival, he had smoked two drops of electronic cigarette fluid from a bottle labelled “VaporFi”, mixed with a transparent liquid from another unlabelled bottle that he found to be “liquid cannabis”. The declared ingredients of the “VaporFi” were propylene glycol, glycerin and natural and artificial flavours; the composition of the unlabelled transparent fluid was unknown. The two bottles were purchased over the Internet and were intended for “vaporization” with an electronic device that aerosolises liquids	Urine	15.6 ng/mL (41 nM)	AB-FUBINACA: 5.6 ng/mL; lidocaine, clindamycin, and cetirizine	Supraventricular tachycardia, mild hypokalaemia, acute confusion, agitation, visual hallucinations, and palpitations. The patient recovered uneventfully with supportive treatment and was discharged 22 h after admission	[4] (2017)
AMB-FUBINACA and ADB-FUBINACA (“Black Mamba”)	Non-fatal; From August 1 to November 30, 2016, eight acute poisoned patients went to the emergency room after consuming “Black Mamba”. There were four men and four women between the ages of 16 and 43	Serum and urine	**Metabolites:** 45.3–115.9 ng/mL in serum and <1.599 ng/mL (LOQ) in urine; **ADB-FUBINACA** < 31.25 ng/mL (LOQ) in serum; **AMB-FUBINACA**: 58.7–115.9 ng/mL in serum	**Cocaine:** 7.8 ng/mL in the serum; 59.2, 2203 and 2362 ng/mL in the urine of 3 different patients; **Benzodiazepine:** 3078 ng/mL in the blood; 25.710 and 29.368 ng/mL in the urine of 2 different patients; ∆^9^-THC; **(Meth) Amphetamine:** 59.5, 349.1, 1461 ng/mL in serum of 3 different patients; 177 and 765.2 ng/mL in urine of 2 different patients; Ethanol; **NGR-3:** <15.6 ng/mL (LOQ) in serum and 11.2 ng/mL in urine; **3-MeO-PCP:** 60.3–114.1 ng/mL in urine	Tonic–clonic seizures, elevated blood pressure. Four patients were agitated and/or delirious. Four patients had chest pain and one had T wave inversions on the electrocardiogram	[60] (2018)
ADB-FUBINACA	Non-fatal; A 38-year-old male inmate was transferred to a medical centre after a seven-day hospitalization for abnormal behaviour (altered mental status and bradycardia). A computed tomography scan of the abdomen and pelvis revealed multiple packages in the patient’s stomach and rectum. Multiple attempts at gastrointestinal decontamination were unsuccessful. On hospital day eight, the patient developed hypertensive emergency and was taken to the operating room for exploratory laparotomy. Twenty-two poorly wrapped packages were removed from the bowel	Serum and urine	34 ng/mL (89.36 nM) in serum; 17 ng/mL (44.68 nM) in urine	Benzodiazepine, ∆^9^-THC-COOH, metoclopramide, atropine, MDMB-FUBINACA, diphenhydramine, metoclopramide, scopolamine and midazolam Cocaine: <20 ng/m (LOQ)	Upon arriving at the treatment unit, the patient was awake, but with inadequate answers to questions, complaining of shortness of breath and staring blankly into space. He demonstrated progressive encephalopathy, second-degree atrioventricular block type I, hypotension, sinus bradycardia, hypoglycaemia, hypothermia, hypopnea, and respiratory failure. Postoperatively, the patient demonstrated both generalised and focal seizure activity. His mental status slowly returned to baseline over the period of about one week and he was ultimately discharged without neurological sequelae after one month	[61] (2018)
“Crystal” and ADB-FUBINACA	Fatal; According to the mother, her 23-year-old son consumed “crystal” at night with friends and arrived home at 4 a.m., feeling bad and going to bed. When she entered the room, the son was kneeling on the bed, so he leaned forward, lost consciousness and died at 9:30 am. When the ambulance arrived, they tried to revive him, without success. The general practitioner declared death and suspected poisoning. The police found no illicit or designer drugs in his room	Blood	ADB-FUBINACA: 0.08 ng/mL (0.21 nM)	**NEH:** 285 ng/mL (749 nM)	Tachycardia, acute heart and pulmonary failure	[62] (2019)
AMB-FUBINACA	Fatal; A 27-year-old man was found dead in his bed by a roommate at about 11 a.m. He had been last seen alive at around 5 a.m.. The doctor checked the body at 2:45 p.m. and found no injuries to the corpse. However, vomiting was observed evolving from the oral cavity and nasal passages. The doctor was unable to determine the cause of death at the scene but found that the man had died about 3 to 8 h earlier. Empty alcohol bottles were found in the apartment, and 20 mg omeprazole tablets were found in the corpse. According to the testimonies of family members and roommates, the man had been drinking alcohol daily, for about three years. He had been treated for paranoid schizophrenia. The man started smoking marijuana at age 16 and later became addicted to “legal drugs”. The autopsy was performed five days after death	Blood and urine	**In urine:** 4.7 ng/mL (12.35 nM); and 8.2 ng/mL (21.55 nM) after hydrolysis of metabolites; **In blood:** no drug detected (LOQ ≤ 0.1 ng/mL)	**EMB-FUBINACA:** no drug detected (LOQ ≤ 0.1 ng/mL) in blood: 0.2 ng/mL in urine; **Lorazepam:** 6 ng/mL in blood and 37 ng/mL in urine; **Haloperidol:** 11 ng/mL in blood and 4 ng/mL in urine; **Lidocaine:** 29 ng/mL in blood and 35 ng/mL in urine	Vomit, congestion of internal organs, pulmonary oedema and left-sided pleural adhesions were found in *post-mortem* examination. The cause of death was acute respiratory failure with an unidentifiable cause	[2] (2019)
ADB-FUBINACA	Fatal; A 17-year-old boy died after smoking an unknown product. Soon after consumption, he experienced uncontrollable tremors and vomiting. After 6 h, he entered the emergency room already dead	Peripheral blood (femoral), urine, stomach and biliary contents	**In blood:** 56 ng/mL (146.5 nM)- the largest documented so far	-	Tremors and vomiting. The cause of death was attributed to the toxicity of ADB-FUBINACA since no other substance was found	[92] (2019)

**∆^9^-THC:***trans*-∆^9^-Tetrahydrocannabinol; **∆^9^-THC-COOH:** 11-*nor*-9-Carboxy-∆^9^-tetrahydrocannabinol; **CNS:** Central nervous system; **LOQ:** Limit of quantitation; **NEH:** *N*-Ethyl-hexedrone; **SC:** Synthetic cannabinoid.

## Data Availability

Not applicable.
